# Hyaluronic Acid: Production Strategies, Gel-Forming Properties, and Advances in Drug Delivery Systems

**DOI:** 10.3390/gels11060424

**Published:** 2025-06-01

**Authors:** Maciej Grabowski, Dominika Gmyrek, Maria Żurawska, Anna Trusek

**Affiliations:** Department of Micro, Nano and Bioprocess Engineering, Wroclaw University of Science and Technology, Wybrzeze Wyspianskiego 27, 50-370 Wroclaw, Poland258633@student.pwr.edu.pl (M.Ż.)

**Keywords:** hyaluronic acid, microbial hyaluronic acid production, hyaluronic-acid-based hydrogels, drug delivery

## Abstract

Hyaluronic acid (HA) is a naturally occurring glycosaminoglycan widely recognised for its biocompatibility, biodegradability, and unique viscoelastic properties. Its structural versatility enables the formation of hydrogels with tuneable physicochemical characteristics, making it a valuable biomaterial in drug delivery and regenerative medicine. This review outlines HA properties, gel-forming approaches, and modern medicine and bioengineering applications. It provides a comprehensive overview of advances in HA production strategies, including microbial fermentation, animal tissue extraction, and production in vitro. Particular attention is given to gel-forming mechanisms, emphasising physical and chemical crosslinking methods like carbodiimide crosslinking, radical polymerisation, and enzymatic crosslinking. Advances in HA-based drug delivery systems and applications of HA-based materials in tissue engineering are also discussed, focusing on HA-based hydrogels with conjugates and combinations with compounds like collagen, alginate, and chitosan.

## 1. Introduction

The earliest documented reference to hyaluronic acid (HA) dates back to 1880, when French chemist Pierre Portes noted that the mucin in the vitreous body exhibited distinct properties compared to other mucoid substances found in the cornea and cartilage. He termed this unique compound “hyalomucin” [1]. In an article published in the *Zentralblatt für praktische Augenheilkunde*, he presented the results of his research on the chemical composition of the vitreous body of the eye, with a particular focus on the presence of hyalomucin. He conducted a detailed biochemical and histological investigation, identifying key constituents and cellular structures. His research emphasised the role of hyalomucin as the principal component responsible for the gel-like consistency and transparency of the vitreous. Through careful chemical analysis, Portes quantified the following substances in the vitreous humour: hyalomucin (0.75‰), albumin (0.27‰), and globulin (0.90‰).

He identified hyalomucin as a mucopolysaccharide–protein complex that is a predominant compound, contributing significantly to the physical properties of the vitreous body, particularly its viscosity and optical clarity. Histological examination revealed that the inner limiting membrane (hyaloid membrane) lacks an epithelial lining, contradicting earlier assumptions of its cellular coverage. Moreover, Portes observed scattered cellular elements predominantly located in the region of the Zonula Zinnii (ciliary zonule). Based on their morphology and arrangement, these cells were interpreted not as leukocytes or inflammatory cells but as residual embryonic mesodermal elements. Their alignment in parallel with the zonular fibres suggests a developmental rather than pathological origin [2].

It was not until 1934 that K. Meyer and J. Palmer first successfully isolated HA from bovine vitreous humour at Columbia University in New York. In their seminal study, Meyer and Palmer employed a modified version of the earlier protocol developed by Swedish chemist and physician Carl Thore Mörner. Mörner’s method, which involved precipitating fresh vitreous humour with dilute acetic acid, isolated a protein–polysaccharide complex that he classified as a mucoid without further separation of its constituents.

Meyer and Palmer refined this approach by applying milder extraction conditions, including sequential precipitation with cold acetone and alcohol, while deliberately avoiding harsh hydrolytic treatments. This allowed them to isolate the non-proteinaceous fraction of the vitreous humour, primarily composed of uronic acid and amino sugars. Their findings demonstrated that the principal macromolecular component of the vitreous body is not a protein-based mucoid but a distinct, high-molecular-weight acidic polysaccharide—subsequently named hyaluronic acid [3].

**Figure 1:**
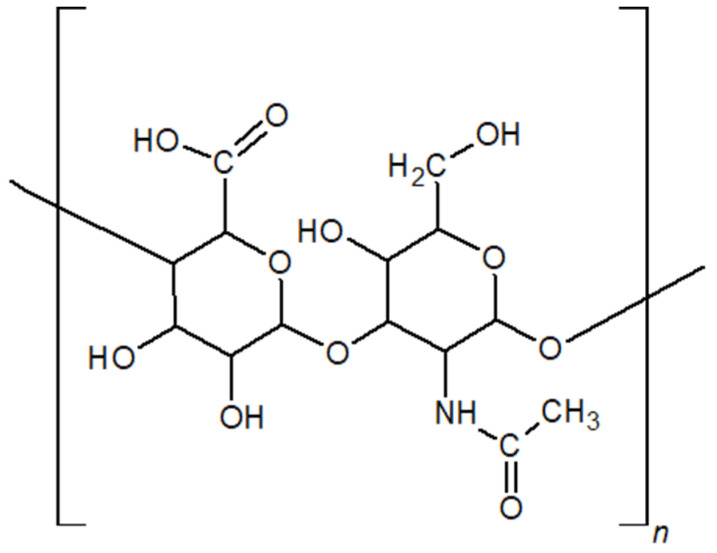
HA structure (d-glucuronic acid and *N*-acetyl-d glucosamine moieties alternate).

The molecular structure of HA was elucidated in 1970 by T.C. Laurent, and in 1989, Endre Balazs introduced the term “hyaluronan” as a scientifically accurate alternative to “hyaluronic acid” [4,5]. Since then, many studies have been conducted in an effort to gain more insight into HA’s properties. This review compiles the information gathered over the years, both old and new, giving special attention to HA production methods and gel-forming approaches, as those items tend to get less spotlight than HA applications. HA is a linear, high-molecular-weight mucopolysaccharide composed of repeating disaccharide units of D-glucuronic acid and N-acetylglucosamine arranged in an alternating sequence (Figure 1) [6]. These monomers are linked by β(1→3) and β(1→4) glycosidic bonds [7]. When both monosaccharides adopt the β-configuration, the resulting structure is highly energetically stable. This is due to the spatial arrangement in which each major functional group (hydroxyl, carboxyl, acetyl, and anomeric carbon) occupies a sterically favourable equatorial position. At the same time, each hydrogen atom resides in the less favourable axial orientation [8]. Consequently, free rotation around the glycosidic bonds within the HA backbone is restricted, resulting in a relatively rigid molecular conformation. In this conformation, nonpolar CH groups alternate with polar groups, which participate in intra- and intermolecular hydrogen bonding, further stabilising the three-dimensional structure of the polymer [9].

At physiological pH, each carboxyl group within HA carries a negative charge, which is counterbalanced by mobile cations such as Na^+^, K^+^, Ca^2^^+^, or Mg^2^^+^. As a result, HA in aqueous solution is negatively charged and forms highly hydrophilic salts, becoming surrounded by water molecules. In solution, HA double helices can organise into duplexes, creating a tertiary β-sheet-like structure. This conformation is stabilised by hydrophobic interactions and intermolecular hydrogen bonding, which promote the aggregation of polymer chains into an extended network.

The formation and stability of this network depend on the molecular weight and concentration of HA. In a recent systematic study performed by Di Meo et al., pharmaceutical-grade HA samples, with molecular weights ranging from 60 kDa to 2.5 MDa, were evaluated across a wide concentration range (0.1–32 g/L). This allowed for detailed characterisation of the material’s rheological properties, thermal and enzymatic stability, and effects on fibroblast bioactivity.

HA solutions displayed distinct rheological profiles based on both molecular weight and concentration. High-molecular-weight HA (≥220 kDa) formed entangled polymer networks, exhibiting pseudoplastic behaviour, particularly the above-defined critical concentrations. These networks were characterised by zero-shear viscosity that increased with molecular weight and concentration, following a power-law scaling. In contrast, low-molecular-weight HA (60–90 kDa) demonstrated Newtonian flow at all tested concentrations, indicating minimal interchain entanglement due to its stiff, rod-like structure. Stability assessments revealed that low-molecular-weight HA is highly resistant to thermal sterilisation and enzymatic hydrolysis by hyaluronidase. This makes it ideal for formulations that require stability under processing or in vivo conditions. In contrast, HA with higher molecular weight showed greater sensitivity to thermal and enzymatic degradation, with depolymerisation increasing with molecular weight and decreasing with concentration [10]. The effect of molecular weight on network formation and viscosity behavior is presented in Table 1.

For instance, native HA with a molecular weight exceeding 10^6^ Da can form an expanded network even at very low concentrations (as low as 1 µg/mL). As both the molecular weight and concentration increase, the resulting HA networks become more robust, leading to enhanced viscosity and viscoelastic behaviour in solution [8,11,12].

Both carbohydrate residues in HA adopt stable chair conformations, which influence the polymer’s overall conformation in solution—often described as a statistical coil with localised regions of high flexibility. Despite this structural variability in solution, from a chemical standpoint, HA is a simple, linear, high-molecular-weight polymer with exceptional rheological properties. It belongs to the glycosaminoglycan (GAG) family, which also includes chondroitin sulphate, keratan sulphate, and heparan sulphate—each characterised by distinct repeating disaccharide units that may be carboxylated or sulphated [13].

HA is unique among glycosaminoglycans (GAGs) in that it is not sulphated and does not form covalent bonds with the core proteins of proteoglycans. Additionally, unlike other GAGs, it is not synthesised within the Golgi apparatus but at the inner surface of the plasma membrane. HA is also capable of reaching exceptionally high molecular weights—up to 10^8^ Da—whereas other GAGs typically range from 1.5 × 10^4^ to 2 × 10^4^ Da [14,15]. This distinctive structural feature enables HA to retain large amounts of water, which imparts pseudoplastic behaviour and gives rise to its viscoelastic properties: high elasticity under high shear rates and high viscosity at low shear rates [15].

As a polyelectrolyte, the rheological behaviour of HA in aqueous solutions is also influenced by ionic strength, pH, and temperature. An increase in any of these factors leads to a marked decrease in viscosity, suggesting a weakening of intermolecular interactions between polymer chains. HA is particularly sensitive to pH changes. In both acidic and basic environments, a delicate balance exists between repulsive and attractive forces; when the pH drops below 4 or rises above 11, HA undergoes degradation through hydrolysis. This effect is more pronounced under alkaline conditions, where hydrogen bonds—critical for the structural organisation of HA chains—are disrupted.

Under strongly acidic conditions, HA undergoes acid-catalysed hydrolysis, which cleaves its glycosidic linkages. This reaction leads to depolymerisation and a significant reduction in the molecular weight of the polymer. Hydrolysis occurs primarily through protonation of the glycosidic oxygen, rendering the bond susceptible to cleavage. Although this process is relatively slow compared to alkaline degradation, it still causes measurable losses in viscosity and elasticity over time. Experimental studies have shown that exposure of HA to pH values below four results in fragmentation of the polysaccharide chains, decreasing solution viscosity and losing its characteristic viscoelastic gel behaviour [16,17]. Hyaluronic acid is significantly more susceptible to degradation in alkaline environments than acidic ones. At pH levels above 11, the primary degradation mechanism is β-elimination, a base-catalysed reaction that cleaves the glycosidic linkage between the glucuronic acid and N-acetylglucosamine residues. This reaction proceeds rapidly and irreversibly, dramatically reducing molecular weight and a complete breakdown of HA’s polymer backbone. In addition to chain scission, exposure to high pH leads to the deprotonation of carboxylic acid groups on the glucuronic acid residues. This increases electrostatic repulsion between negatively charged HA chains, causing them to expand and lose structural cohesion. Furthermore, hydrogen bonding, which stabilises the entangled or cross-linked network of HA chains in gel form, is significantly disrupted under these conditions. The combined effect of backbone cleavage and loss of intermolecular interactions leads to a collapse of the three-dimensional gel structure [16,17].

Nečas et al. noted that degradation under alkaline conditions is more severe and irreversible, resulting in an abrupt and sustained drop in molecular integrity and rheological performance. This phenomenon is particularly critical in applications requiring long-term structural stability, such as intra-articular injections and dermal fillers [16].

Thus, both the structural characteristics and the polyelectrolytic nature of HA are key determinants of its rheological profile [1,13,14,15]. HA solutions exhibit non-Newtonian fluid behaviour. Their pseudoplastic profile arises from the breakdown of intermolecular hydrogen bonds and hydrophobic interactions under increasing shear rates. During flow, HA chains deform and align along streamlines, resulting in a noticeable decrease in viscosity [18]. Additionally, HA solutions are non-thixotropic—they fully regain their original structure and viscosity once the shear rate decreases, following the same intermediate states experienced during the breakdown process. This means the polymer network disassembly is both transient and reversible. Such distinctive rheological behaviour is crucial, as it underpins virtually all of HA’s biomedical and biotechnological applications [19].

The rheological behaviour of HA as a polyelectrolyte in aqueous solutions is affected by ionic strength, and pH ionic strength is critical in determining HA conformation in solutions. At low ionic strength, unscreened electrostatic repulsion causes the polymer chains to adopt extended conformations, resulting in increased hydrodynamic volume and elevated viscosity. Upon addition of salt, screening these repulsive interactions induces chain contraction, leading to a decrease in the radius of gyration and viscosity. This behaviour is characteristic of semirigid polyelectrolytes and is well captured by Monte Carlo simulations, which align with experimental light scattering data.

pH exerts a dual influence on HA by altering its ionisation state and structural stability. The polymer remains stable within the approximate pH range of 5–10. Outside this window, degradation occurs: acid-catalysed hydrolysis dominates at low pH, while β-elimination is the prevailing mechanism under strongly alkaline conditions (pH > 11). Both processes result in chain scission, reduced molecular weight, and a consequent loss of viscoelasticity. HA solutions approach Newtonian flow behaviour at extreme pH values, particularly pH 13, due to the breakdown of intermolecular associations. Under neutral pH, temperature exerts a comparatively minor influence on HA’s rheological behaviour. Structural studies using small-angle neutron scattering indicate minimal changes in chain conformation between 25 °C and 37 °C, suggesting that thermal effects on hydrogen bonding and entanglement are negligible under physiological conditions. However, elevated temperature may accelerate pH-dependent degradation kinetics, indirectly affecting viscosity over time [20].

HA exerts its biological effects via two fundamental mechanisms: as a passive structural molecule and as an active signalling molecule. Both modes of action are strongly dependent on the molecular weight of HA. The passive mechanism is primarily associated with high-molecular-weight HA, which—owing to its large size, pronounced hygroscopicity, and viscoelastic properties—can modulate tissue hydration, osmotic balance, and the physical properties of the extracellular matrix (ECM). In this role, HA contributes to the formation of a hydrated and stable extracellular space, where cells, collagen fibres, elastin, and other ECM components are structurally supported [21].

HA also functions as a signalling molecule by interacting with HA-binding proteins. These interactions depend on several variables, including HA’s molecular weight; tissue localisation; and cell-specific factors such as receptor expression, signal transduction pathways, and the cell cycle stage. Depending on these conditions, HA–protein binding can elicit opposite biological effects: pro-inflammatory or anti-inflammatory responses, cell migration promotion or inhibition, and stimulation or suppression of cell proliferation and differentiation. These outcomes can be modulated by HA’s molecular weight, which also affects cellular uptake and receptor binding affinity. HA-binding proteins are generally categorised into two groups: matrix-associated HA-binding proteoglycans (also known as extracellular hyaladherins) and cell-surface HA receptors (cellular hyaladherins) [1,22]. HA engages with hyaladherins through two main molecular mechanisms. First, it can act autocrine by binding receptors on the same cell that produced it [23]. Second, it can behave as a paracrine by interacting with receptors on neighbouring cells, activating various intracellular signalling cascades. High-molecular-weight HA can simultaneously bind multiple receptors on a single cell surface and interact with several proteoglycans. These large complexes can, in turn, associate with additional ECM proteins, forming supramolecular structures that anchor to the cell surface via HA receptors [24].

Thus, HA serves as a molecular scaffold that not only stabilises ECM architecture through its passive structural role but also actively regulates ECM dynamics via interactions with extracellular hyaladherins such as aggrecan, neurocan, and versican. This dual functionality highlights HA’s central role in maintaining the integrity and functionality of connective tissues and protecting them from environmental stressors [25].

Interactions between HA and cell surface receptors are crucial in three key biological processes: signal transduction, forming pericellular matrices, and receptor-mediated endocytosis. Notable receptors that mediate these processes include CD44, RHAMM, TSG-6, LYVE-1, and HARE [1,26].

RHAMM (receptor for HA-mediated motility) and CD44 have garnered the most scientific attention. CD44 is a structurally diverse and multifunctional transmembrane glycoprotein expressed on the surface of most cell types. It is currently considered the principal HA receptor across a wide range of tissues and is the most extensively characterised HA-binding receptor [27]. CD44 mediates various cellular processes, including adhesion, migration, and signal transduction, by interacting with extracellular HA and intracellular cytoskeletal components. RHAMM is found not only on the cell surface but also in the cytosol and nucleus. This receptor is critically involved in regulating cellular responses to growth factors and plays a central role in cell migration, particularly in fibroblasts and smooth muscle cells [28]. The HA receptor for endocytosis (HARE), initially isolated from endothelial cells of the liver, lymph nodes, and spleen, has also been identified in endothelial cells of the eye, brain, kidney, and heart. HARE is capable of binding HA and other glycosaminoglycans (GAGs), except for keratan sulphate, heparan sulphate, and heparin. Its primary function involves the clearance of GAGs from circulation. LYVE-1 (lymphatic vessel endothelial HA receptor 1) is another HA-binding protein expressed predominantly in lymphatic endothelial cells and specific macrophage populations. LYVE-1 mediates the uptake of HA from tissues into the lymphatic system, thereby regulating HA turnover and transport [18].

Toll-like receptors (TLRs) act as key activators of monocytes, macrophages, and dendritic cells, making them essential initiators of the immune defence against bacterial infections [29]. During inflammation or tissue injury, high-molecular-weight HA in the extracellular matrix is degraded into smaller fragments. These low-molecular-weight (LMW) HA fragments can trigger an innate immune response.

Research by Scheibner et al. demonstrated that this immune activation is due to the binding of LMW HA fragments to Toll-like receptors, particularly TLR-2 and TLR-4 [30]. These interactions initiate signalling cascades that promote inflammation and recruit immune cells to the site of damage.

Moreover, LMW HA degradation products have been found to contribute to scar formation, indicating that not only the molecular weight but HA itself plays an active role in wound healing and scar development. While high-molecular-weight HA (HMW-HA) helps maintain tissue integrity and suppresses inflammation, its shorter fragments are perceived as a signal of tissue damage, thus triggering a pro-inflammatory response [31]. HMW-H, typically found in the healthy extracellular matrix, promotes tissue stability and suppresses inflammation primarily through CD44 cross-linking, which facilitates anti-inflammatory signalling, enhances regulatory T cell function (e.g., Foxp3 expression and IL-10 production), and inhibits Toll-like receptor-mediated pathways. Conversely, during tissue injury or oxidative stress, HA is enzymatically degraded into low-molecular-weight fragments (LMW-HA), which function as damage-associated molecular patterns. These fragments are recognised by TLR2 and TLR4, triggering NF-κB activation and upregulation of pro-inflammatory cytokines such as TNF-α, IL-1β, and IL-6. The inability of LMW-HA to cross-link CD44 distinguishes it from HMW-HA, thereby shifting the cellular response from homeostasis to inflammation. This size-specific signalling system enables HA to act as a contextual biosensor differentiating between intact and injured tissue environments [32].

HA occurs naturally as a hydrated gel and is ubiquitous in the tissues of both humans and animals. Despite its relatively simple structure, one might mistakenly conclude that it limits the range of its biological functions. However, the multitude of proteins and receptors that bind HA, its widespread presence, and its biological and chemical properties determine the richness of its interactions within the body. This macromolecule influences cell behaviour and plays significant structural, rheological, physiological, and biological roles in the organism [33,34,35].

## 2. HA Production

For many years, HA was extracted exclusively from animal tissues—most notably from rooster combs (with concentrations reaching up to 7500 μg/g), but also from bovine vitreous humour and synovial fluid. However, bacterial fermentation and in vitro methods are increasingly used for HA production due to ethical, safety, and scalability considerations [9,36]. The general reaction for HA synthesis is as follows:

n UDP-GlcUA + n UDP-GlcNAc → 2n UDP + [GlcUA + GlcNAc]_n_

This reaction is catalysed by a single enzyme that uses both sugar substrates to form the repeating disaccharide unit of HA.

**Figure 2:**
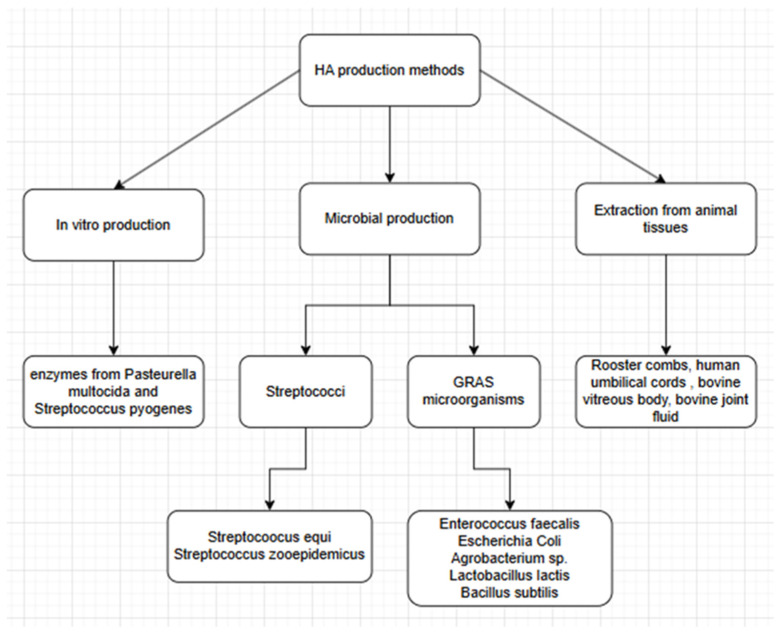
HA production pathways.

We can distinguish three ways of obtaining HA (Figure 2): extraction from animal tissues, microbial production, and production in vitro. The main differences between these methods are summarized in Table 2.

### 2.1. Extraction from Animal Tissue

The extraction of HA from animal tissues was initially used for laboratory research to identify and characterise the polymer and to explore its biological potential and biomedical applications. HA has been isolated and described from almost all vertebrate tissues, including the vitreous body of the eye, umbilical cord, synovial fluid, pig skin, rabbit pericardial fluid, and shark cartilage. Recently, HA has been extracted from fish eyes as an alternative source outside animal farming. However, the most accessible sources of high-molecular-weight HA for large-scale production are rooster combs [38,39]. In 1979, Balazs developed an effective procedure for isolating and purifying HA from rooster combs and human umbilical cords, which laid the foundation for the industrial production of HA from rooster combs for medical applications [40]. HA is water-soluble, but the extraction of highly pure HA with high molecular weight from animal tissues is challenging, as HA is usually present in complexes with other biopolymers, including proteoglycans [41]. Methods for releasing HA from these complexes include enzymatic treatment, precipitation of hyaluronic ions (e.g., using cetylpyridinium chloride), precipitation with organic solvents, non-solvent systems, detergents, and more. Ultrafiltration and chromatography are used to remove degradation products and other contaminants [42]. However, the extraction of HA from animal tissues is gradually being abandoned due to various limitations.

Additionally, the fact that animal-derived HA may still be bound to cellular proteins, such as hyaluronidase, increases the popularity of microbiological production. The presence of contaminating proteins in HA poses a risk of immunological reactions. Furthermore, HA obtained from animal tissues may be contaminated with prions and viruses, which could result in disease transmission [43].

An exemplary procedure for extracting HA from rooster combs was described in 2010 by Kang and Lee in the *Journal of Separation Science*. Initially, 500 g of frozen rooster combs were mechanically homogenised using an electric grinder, yielding tissue fragments approximately 0.5 cm in size. These fragments were subjected to defatting by immersion in 1 L of acetone at refrigeration temperature (approximately 4 °C). The acetone was decanted after 2 h and replaced with fresh acetone. This defatting step was repeated ten times. Following the final wash, residual acetone was removed by air-drying. The resulting dried, defatted material weighed approximately 80 g. Subsequently, the defatted tissue was subjected to sequential aqueous extraction. The material was extracted ten times with 1 L aliquots of 5% sodium acetate solution (w/v), with each extraction conducted over a 2 h interval.

After each extraction, the viscous supernatant was recovered by manual compression through a cotton cloth. The remaining solid residue was discarded after the final extraction. To precipitate HA, 1.5 L of ethanol was added to the pooled aqueous extracts. The precipitated material was collected by centrifugation, redissolved in 5% sodium acetate solution, and centrifuged again. Proteinaceous contaminants were removed by successive liquid–liquid extraction steps, first with 100 mL chloroform (four times), followed by chloroform–amyl alcohol mixture (1:2 v/v) repeated until no gel formation was observed. The purified supernatant was dialyzed against distilled water. HA was reprecipitated with ethanol and subsequently lyophilised to yield a dry powder. The final yield of HA was approximately 500 mg. To prepare an aqueous HA solution, 50 mg of the lyophilised HA powder was dispersed in 15 mL of distilled water and heated on a hotplate at 30–40 °C for 15 min under constant stirring. The volume was then adjusted to 50 mL with additional distilled water. The HA dissolved wholly but slowly, forming a clear, viscous solution [44].

### 2.2. Enzymatic Synthesis

The synthesis of HA using isolated HA synthase becomes important when polymers of HA with a specific molecular weight and narrow polydispersity are required. The isolated HA synthase can catalyse the same reaction in vitro under strictly defined conditions as it does in vivo, namely, the synthesis of HA from nucleotide sugars UDP-GlcNAc and UDP-GlcUA. Enzymatic synthesis of HA has been demonstrated using membrane-bound HA synthase from *Streptococcus pyogenes*, although the yield was low, about 20% [45]. The yield of HA increased to 90% when enzymatic HA synthesis was combined with the enzymatic regeneration of sugar nucleotides in situ using UDP and relatively inexpensive substrates, Glc-1-P and GlcNAc-1-P, in a one-pot reaction. The average molecular weight of the synthetic HA was about 5.5 × 10^5^ Da. Control of the chain length and polydispersity of the HA polymer is determined by the inherent enzymatic properties of the recombinant PmHAS (operon containing HA synthase from *Pasteurella multocida*). The rate-limiting step of in vitro polymerisation is chain initiation. Since in vitro enzymatic polymerisation is a fast, non-processive reaction, the HA acceptor’s concentration controls the HA polymer’s size and polydispersity in the presence of a finite amount of UDP sugar monomers [46]. Although in vitro production appears to hold significant potential, numerous technological problems must first be solved. It is necessary to develop an effective and relatively inexpensive method for selectively separating the UDP by-product from the reaction mixture to prevent enzyme inactivation and enable UDP recycling. Efficient processes for producing HA matrices for elongation must also be developed, along with simple, cost-effective technologies for synthesising sugar nucleotide substrates [47].

Because Class I HA synthases are integral membrane proteins, their use in in vitro production of HA is limited by the complex and labour-intensive isolation procedures and their dependence on close association with the phospholipid bilayer for proper functionality. In contrast, the peripherally membrane-associated Class II HA synthases from *Pasteurella multocida* present a more viable option for in vitro HA synthesis at a commercial scale. Deletion of the membrane-spanning residues 704–972 from the Class II enzyme yields a truncated, soluble variant, referred to as pmHAS_1-703_, which retains catalytic activity for HA production. In vitro (cell-free) production systems utilising this Class II-derived synthase have demonstrated the ability to generate HA polymers with molecular weights of approximately 1–2 MDa. These systems also offer low polydispersity and controllable polymer characteristics by adding HA oligomers, facilitating tailored processability [48,49].

### 2.3. Microbial Production

Since HA synthesised by animals and bacteria is structurally identical, bacterial-derived HA is non-immunogenic and thus represents an excellent source of medical-grade HA. The extraction of HA from microbial fermentation broth is a relatively straightforward and highly efficient process. A key advantage of microbial production is the ability to physiologically and/or metabolically engineer microbial cells to enhance the yield of high-molecular-weight HA, i.e., native strains of *Streptococcus zooepidemicus* naturally produce HA with molecular weights typically ranging from 1 to 3 MDa. While effective, these native systems are constrained by their metabolic tendencies, such as lactic acid accumulation, which can reduce yield and polymer size. However, even minor adjustments in fermentation conditions—such as nutrient substitution or pH regulation—can influence Mw. Benedini and Santana demonstrated that replacing animal-derived brain heart infusion with soy peptone increased HA molecular weight from approximately 3.1 MDa to 3.6 MDa, likely due to decreased lactic acid stress and better pH stability [50]. Although initial yields and Mw in recombinant systems tend to be lower (typically 0.5–1.5 MDa), metabolic engineering can improve both. Recombinant *B. subtilis* strains have been optimised to produce HA exceeding 2 MDa, with reduced endotoxin risk due to their GRAS status [33,51].

Consequently, microbial synthesis of HA using either pathogenic *Streptococci* or generally recognised as safe (GRAS) organisms such as *Lactococcus lactis* or *Bacillus subtilis* has become increasingly common [9]. The first documented isolation of HA from group A haemolytic *Streptococci* yielded 60–140 mg/L of HA. Over the past three decades, substantial improvements in production technology have led to a current batch fermentation yield of approximately 6–7 g/L, representing a practical upper limit due to mass transfer constraints [52].

Group C *Streptococci,* which are non-pathogenic to humans, exhibit higher HA productivity and are therefore frequently employed in place of group A *Streptococci* or *Pasteurella multocida*. The most commonly utilised strains include *Streptococcus equi* subsp. *equi* and *Streptococcus equi* subsp. *zooepidemicus* [53,54].

#### 2.3.1. *Streptococcus* sp.

Gram-positive *Streptococci* are non-sporulating, non-motile bacteria. Various wild strains, such as *Streptococcus equisimilis*, *Streptococcus pyogenes*, and *Streptococcus uberis*, can produce HA. Among them, *S. zooepidemicus* and *S. equisimilis* are the most prominent producers [55].

*S. zooepidemicus* is a commensal and opportunistic mucosal pathogen that infects several animal species (primarily horses) and only rarely affects humans. It is most commonly isolated from the uterus of mares and is a subspecies of *S. equi* [56]. This bacterium possesses an operon responsible for HA synthesis. The operon was first discovered in the early 1990s when the gene encoding the HA-synthesising enzyme was isolated from *S. pyogenes* [43,57]. This operon comprises five genes encoding hyaluronic acid synthase (HasA), UDP-glucose dehydrogenase (HasB), UDP-glucose pyrophosphorylase (HasC), UDP-N-acetylglucosamine pyrophosphorylase (glmU), and phosphoglucose isomerase [43]. Cloning of the genes encoding streptococcal HA synthase suggests that the HasA enzyme alone is sufficient for HA biosynthesis, provided the cell already synthesises glucuronic acid and N-acetylglucosamine [55]. Moreover, HA production efficiency has been increased with overexpression of the HasA enzyme [58].

*S. pyogenes* is also capable of producing HA. However, due to its status as a strictly human pathogen, it is used less frequently than *S. zooepidemicus.* This bacterium is a beta-haemolytic *Streptococcus* belonging to the serological group A and is a known etiological agent of pharyngitis (strep throat) and other diseases such as impetigo and scarlet fever. Like *S. zooepidemicus*, *S. pyogenes* also possesses the operon responsible for HA synthesis [59,60].

#### 2.3.2. *Pasteurella multocida*

*Pasteurella multocida* is a non-motile, Gram-negative *coccobacillus* and a known pathogen responsible for pneumonia in piglets and calves. In this microorganism, HA synthesis is catalysed by a polypeptide encoded by a single gene, pmHAS. This gene encodes the HA synthase enzyme, which polymerises HA by adding glucose-6-phosphate and fructose-6-phosphate units to the non-reducing end of the growing polysaccharide chain. A notable characteristic of pmHAS is its ability to elongate exogenous, short HA chains in vitro, forming high-molecular-weight polymers. However, in vivo, chain elongation is hindered due to steric interference caused by the growing HA polymer [61,62].

Despite its intrinsic ability to produce HA, *P. multocida* has not been employed for industrial-scale HA biosynthesis, likely due to its pathogenic nature [63]. This bacterium is associated with various diseases in wild and domesticated animals, including birds, mammals, and humans. In poultry, it causes fowl cholera; in swine, atrophic rhinitis; and in cattle, respiratory infections. It has also been linked to mass mortality events in saiga antelopes. In humans, infections can result in localised lymphadenopathy, while more severe manifestations may include bacteraemia, osteomyelitis, endocarditis, and even meningitis if the bacteria cross the blood–brain barrier [60,64].

#### 2.3.3. “GRAS” Microorganisms

As HA produced by *Streptococcus* species may be contaminated with exotoxins inherently associated with these pathogenic bacteria, production hosts classified as “GRAS” have been adopted for industrial-scale HA biosynthesis [47]. GRAS organisms must not secrete toxins and must be capable of continuously synthesising the biopolymer to achieve a molecular weight of at least one megadalton (MDa). HA’s molecular weight and purity are key indicators of its quality, with polymers exceeding 0.5 MDa possessing more excellent market value [55].

Genetic engineering can introduce the operon responsible for HA biosynthesis into relatively safe host strains. The recombinant organisms thereby acquire the capacity to produce HA. Among GRAS-designated strains, *Bacillus subtilis* is most commonly employed; however, *Lactococcus lactis*, *Escherichia coli*, and the widely used food-grade *Streptococcus thermophilus* are also considered viable hosts for HA production [65].

##### *Bacillus* *subtillis*

*Bacillus* species have long been leading industrial microorganisms used to produce a wide range of compounds. These organisms are capable of secreting large amounts of products, indicating their highly developed biosynthetic capacity, and they are very economical to cultivate and have excellent growth capabilities in industrial fermenters. *B. subtilis* is one of the best-characterised Gram-positive microorganisms. Furthermore, its genome has been sequenced, and extensive genetic manipulation tools are available. Importantly, *B. subtilis* is free from exotoxins and endotoxins. This microorganism does not naturally produce HA but acquires this ability through genetic modification. For this purpose, an expression system is used, incorporating the hasA gene from *S. equisimilis* in combination with the overexpression of one or more of the three native precursor genes of B. subtilis (homologous to hasB, hasC, and hasD in *Streptococcus*) [33]. The HA concentration produced by recombinant *B. subtilis* was comparable to that of attenuated streptococci. The success of HA production in *B. subtilis* suggests that genetic engineering provides new possibilities for producing capsular polysaccharides in a heterologous host [57,66]. The biotechnology company Novozymes developed the process for producing ultra-pure sodium hyaluronate through the fermentation of a new, non-pathogenic recombinant *Bacillus subtilis* strain. The recombinant *B. subtilis* strain produced up to 5 g/L of HA with a molecular weight of 1–1.2 million Da when cultured in a minimal sucrose-based medium at pH 7 and 37 °C [67].

##### *Lactococcus* *lactis*

*Lactococcus* is a genus of Gram-positive lactic acid bacteria widely used in the food industry for the production and preservation of fermented products. Due to their GRAS status, genetic engineering efforts have been undertaken to enhance existing traits or confer entirely new functionalities to *Lactococcus* subspecies. One prominent tool in this field is the nisin-controlled expression system (NICE), a well-characterised, versatile, and tightly regulated gene expression system based on the autoregulatory mechanism of the bacteriocin nisin [68,69]

This system enables the construction of plasmids that function in GRAS-grade bacteria and drive the expression of genes from the has operon, including hasA (HA synthase) and hasB (UDP-glucose dehydrogenase). Through this approach, a nisin-negative strain of *L. lactis* can produce HA. Co-expression of hasA, hasB, and hasC from *S. zooepidemicus* in *L. lactis* has doubled the production of HA to 0.243 g/L when compared to only hasA and hasB expression, which yielded 0.107 g/L. When the higher-yielding strain was grown in an aerated bioreactor, the yield of HA went up to 1.8 g/L. Since the host strain *L. lactis* is a food-grade lactic acid bacterium, the resulting HA holds great potential for applications across various industries, particularly in the food and biomedical sectors [70,71]

##### *Escherichia* *coli*

While HA production is commonly observed in Gram-positive bacteria, many Gram-negative bacteria, such as *Escherichia coli*, do not produce HA because they either lack key enzymes in the biosynthetic pathway or maintain the concentration of pathway components at low levels. Therefore, most *E. coli* strains commonly used in laboratories, such as JM109, do not synthesise HA. However, if *E. coli* UDP-glucose dehydrogenase and the pmHAS from *P. multocida* are expressed, HA can be synthesised in *Escherichia coli* strains [72]. HA production in this system, combined with the supplementation of glucose and glucosamine—precursors of HA—during the induction process, resulted in a yield as high as 3.8 g/L and a maximum molecular weight of 1.5 MDa [44]. Through the co-expression of the HA synthase gene hasA derived from *S. equi* or *P. multocida* with the homolog hasB (UDP-glucose dehydrogenase) or hasB + hasC (UDP-glucose dehydrogenase and UDP-glucose pyrophosphorylase) from *E. coli*, improved heterologous strains were obtained. The recombinant *Escherichia coli* strain generated using this strategy produced 2 g/L of HA, and the yield increased to 3.8 g/L when the culture medium was supplemented with glucosamine [55].

##### *Streptococcus* *thermophilus*

*Streptococcus thermophilus* is a Gram-positive, thermophilic lactic acid bacterium with an optimal growth temperature of 45 °C. It belongs to the salivarius group, also known as green-staining *streptococci*, and importantly, it is considered non-pathogenic [73]. The bacterium is exceptionally well-adapted to grow in dairy products. This organism can produce HA from lactose. Izawa et al. demonstrated that this bacterium produces HA in a milk-based medium supplemented with soy peptides as a nitrogen source [74]. Similarly, Zhang and colleagues synthesised HA using *S. thermophilus* metabolising skimmed milk powder supplemented with various carbohydrates. This method yielded HA chains with a molecular weight of 2000 kDa [69]. In 2011, a recombinant *S. thermophilus* was constructed capable of producing 1.2 g of HA per litre, and the average molecular weight of the produced HA was comparable to that of the wild-type strain [73].

#### 2.3.4. Other Microorganism

Additionally, certain eukaryotic microorganisms exhibit the capacity to synthesise HA. For instance, green algae of the genus *Chlorella* can produce HA upon infection with *chloroviruses* or *Paramecium bursaria* viruses. However, the HA yield from *Chlorella* is relatively low (~1 g/L) and does not match the production levels achieved using *streptococcal* fermentation processes [55]. *Cryptococcus neoformans*, a yeast species capable of HA synthesis, is not utilised for commercial production due to its pathogenicity—it is an opportunistic human pathogen responsible for cryptococcosis, a severe fungal infection [65].

Notably, HAS enzymes produced by *streptococci* and vertebrates belong to Class I HAS. In contrast, the enzyme from *P multocida* represents the sole known member of Class II HAS [43].

*Pichia pastoris*, a methylotrophic yeast, has emerged as a promising non-pathogenic platform for the recombinant production of HA. Leveraging its well-characterised expression system and ability to perform eukaryotic post-translational modifications, *P. pastoris* has been genetically engineered to express membrane-bound HAS enzymes from *P. multocida*.

This system offers several advantages over traditional bacterial platforms, including the absence of endotoxins, making it highly suitable for pharmaceutical and cosmetic-grade HA production. Reported conversion efficiencies range from 20 to 90%, depending on fermentation conditions and enzyme activity. However, the HA produced tends to have lower molecular weight (up to ~2.5 MDa) and broader polydispersity than to products derived from *S. zooepidemicus* [37]. As shown in Table 3, HA properties vary depending on the source.

## 3. Bioreactors for HA Production

**Figure 3:**
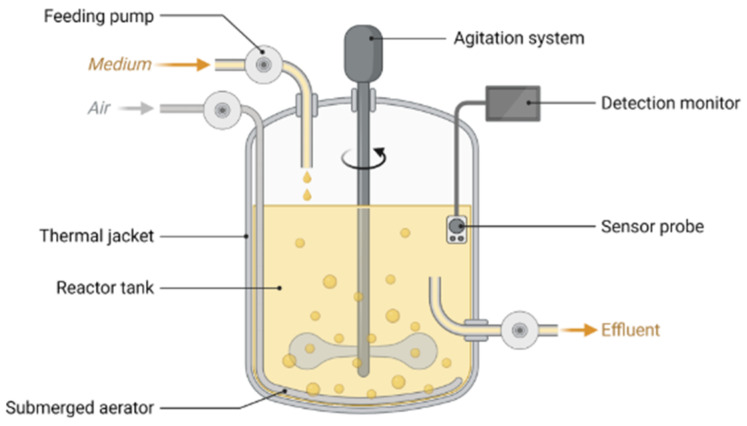
A stirred-tank bioreactor equipped with an aeration system, a thermal jacket, and integrated sensors [83].

Several factors significantly influence the production of HA by microorganisms. These include pH, temperature, agitation rate, aeration rate, and the type of bioreactor used. The use of barbotage bioreactors is generally unsuitable for HA production due to the significant increase in medium viscosity during the process. These bioreactors are most effective in low-viscosity fermentation systems. Similarly, airlift bioreactors present limitations, as their mixing efficiency is suboptimal, leading to local concentration gradients. Inadequate oxygen dispersion in such systems can result in oxidative stress in the microbial culture [82]. Among the various bioreactor systems, stirred-tank bioreactors (Figure 3) are predominantly employed for HA production. These bioreactors are specifically designed to maximise the biological activity of living cells by ensuring optimal interactions between the cells and the culture medium.

In stirred-tank bioreactors, bubble uniformity and dispersion are achieved through mechanical agitation, which requires relatively high energy consumption per unit volume. Typically, 70–80% of the total reactor volume is filled with liquid, leaving headspace for gas exchange and foam formation, which can be managed using a foam breaker [84].

This bioreactor’s key and defining component is the impeller, which performs a wide range of critical functions. These include heat and mass transfer, uniform oxygen distribution, and mixing to ensure the homogenisation of the reactor contents. The most commonly used types of impellers are axial-flow and radial-flow impellers [85].

The extensive understanding and widespread use of stirred-tank reactors facilitate the design and optimisation of entire bioprocess systems. The proper selection of impellers depends not only on the bioreactor dimensions, medium composition, and total working volume but also on the density and viscosity of the liquid being mixed. The application of different impeller types is guided by the viscosity scale of the culture medium [84].

Conventional impellers that generate high shear stress cannot be directly applied in shear-sensitive biological systems, such as cell cultures. Two primary mechanisms are responsible for potential cell damage: hydrodynamic shear forces induced by mixing and mechanical injury caused by air bubbles due to improper gas dispersion. Extensive research has been devoted to eliminating or at least minimising the frequency of cell damage resulting from these mechanisms. A key strategy has been modifying existing impeller designs and developing new, gentler types tailored for sensitive cultures. Additionally, implementing appropriate aeration systems—such as bubble-free aeration—and using protective agents like poloxamers are recommended to reduce cellular stress further and enhance viability [86].

### 3.1. Increase in Viscosity

As with the fermentation of most polysaccharides, the broth used for HA production exhibits non-Newtonian fluid characteristics. The accumulation of HA causes the broth to become excessively viscous, which significantly hampers effective mixing and aeration—critical issues during fermentation. As viscosity increases, the rate of oxygen transfer decreases, reaching its minimum when maximum viscosity is attained. This rise in viscosity necessitates an increase in the impeller’s rotational speed, consequently exposing cells to higher shear stress and mechanical damage [87]. Elevated viscosity limits overall product yield, rendering the production of HA at concentrations above 4–10 g/L impractical and economically inefficient [88,89,90]. Therefore, it is essential to develop processes that allow for continuous removal of HA during fermentation.

### 3.2. Mixing

As the fermentation broth’s viscosity increases, oxygen dispersion becomes increasingly complex, and shear stress rises accordingly. To counteract this effect and enhance dissolved oxygen concentration, it is often necessary to adjust the impeller speed. However, higher impeller speeds result in elevated shear stress, which can damage the cells involved in the process [91]. In Newtonian fluids, the relationship between shear stress and shear rate is linear and passes through the origin, with the proportionality constant being the viscosity. In contrast, non-Newtonian fluids display a nonlinear relationship between these parameters [92].

High shear stress has been shown to slow down HA synthesis and reduce its molecular weight, though it does not significantly affect overall HA yield [91]. Interestingly, Nickel et al. reported that HA chain initiation or elongation was stimulated when the polymer detached from the synthase and was released into the medium. This suggests high shear stress may facilitate HA production by promoting molecules release [93].

Kim et al. observed that the aeration rate did not significantly influence HA production, whereas increasing impeller speed led to a decline in yield. Conversely, Hasegawa and colleagues found that increasing impeller speed enhanced HA production [93]. These conflicting results underscore the critical importance of mixing conditions in HA fermentation.

Researchers have focused on developing and optimising impeller designs to address these challenges. Numerous low-power, high-flow impellers have been engineered to ensure efficient mixing at lower rotational speeds. The issue remains unresolved despite advances and the introduction of various impellers such as Intermig, Maxflow, Maxblend, and Rushton turbines [94].

### 3.3. Aeration

Oxygen is vital in aerobic fermentations, particularly in systems involving non-Newtonian fluids such as those used for HA production [95]. Due to the inherently low solubility of oxygen in aqueous solutions, ensuring an adequate oxygen supply to the bioreactor is essential to prevent oxygen-limited cellular growth. However, the high viscosity of HA fermentation broths can hinder oxygen transfer, making it crucial to understand the relationship between oxygen uptake and transfer rates. Despite its significance, only a few studies have focused on oxygen transfer and uptake parameters during HA fermentation [82]. Given the non-Newtonian nature of HA broths, modulation of agitation and aeration rates remains the primary method for increasing dissolved oxygen (DO) concentrations. Low impeller speeds typically fail to meet the cells’ oxygen demand, whereas high speeds may cause excessive shear stress, leading to cellular damage [88]. Since mixing and aeration effects are often intertwined, studying the independent impacts of DO and shear stress on HA fermentation is essential. Such investigations could yield valuable insights for process optimisation and scale-up [96].

## 4. Process Modes

HA production systems include batch (discontinuous), fed-batch (semi-continuous), and continuous modes.

### 4.1. Batch

The periodic system, also called the batch system, involves the cyclical repetition of production processes. Substrates are supplied to the system at specific intervals, and products and intermediates are harvested after the completion of the production cycle. The process occurs under unsteady-state conditions. Periodic fermentation is the standard mode for HA production. The advantages of a batch reactor stem from its versatility. In periodic reactors, products typically release or absorb heat during processing, and even mixing the stored liquid generates heat. To maintain the reactor content at the desired temperature, heat must be added or removed via a thermal jacket or cooling coil. Another advantage of the periodic reactor is its high conversion rate per unit volume per pass, as well as the ability to produce a single product at a time. However, the main drawback of periodic mode is the lengthy process duration, significantly reducing volumetric production efficiency and resulting in a higher fixed cost per product unit [97,98,99,100,101,102]. According to the data reported by Long Liu et al., the HA production yield in a batch system using *S. zooepidemicus* reached 5.0 g/L [79]

### 4.2. Continuous

A continuous system is one in which substrates are continuously fed into the bioreactor while products are simultaneously removed. The process operates under steady-state conditions. Continuous operation is typically performed using continuous stirred-tank reactors (CSTRs), which have two operating strategies.

The first strategy is the chemostat, commonly used in cell culture processes. In this mode, all nutrients are supplied in excess, and the liquid volume is maintained constant by balancing the inflow and outflow rates. This enables the maintenance of a constant concentration of a specific limiting substrate. The second strategy is the turbidostat, in which cell concentration is kept steady by monitoring and controlling the culture medium’s turbidity (or optical density). As in the chemostat, the reactor volume remains constant by maintaining equal inlet and outlet flow rates. CSTR systems can also be operated in series, with multiple reactors connected sequentially, each operating under different conditions [96,103].

Despite their theoretical advantages, CSTRs are rarely used for HA production due to challenges such as back-mixing and alternating residence times, which have a negative impact on product yield, selectivity, and efficiency per time unit and reactor volume [96]. Due to numerous challenges and the specific nature of HA production, data on HA yield in this system are not available.

### 4.3. Fed-Batch

The semi-continuous system is a hybrid of batch and continuous systems. This approach eliminates the stationary phase of microbial growth [99]. Semi-continuous cultures are achieved by repeating batch processes repeatedly [82]. Reactors operating in a semi-continuous mode function similarly to batch reactors, as the process occurs in a single stirred tank with comparable equipment. However, they are modified to allow for the addition of reagents and removal of products during the process. In a standard batch reactor, all reagents are added at the beginning and the reaction proceeds without further intervention. In contrast, a semi-continuous reactor allows partial filling of the reaction vessel and permits the incremental addition of substrates or other materials over time. This operational “flexibility” offers several advantages that support the superiority of this system over the batch mode. Moreover, it allows product withdraw and improves reaction selectivity. Mixing in these bioreactors is highly efficient, enabling uniform conditions throughout the reactor volume [98]. The HA production yield in the fed-batch system is slightly lower than in the batch system (4.72 g/L in fed-batch, 5.0 g/L in batch); however, the cell concentration increased from 13.3 g/L in batch mode to 14.7 g/L in fed-batch mode [104].

## 5. HA Gel-Forming Strategies

### 5.1. Chemical Modification of HA Functional Groups

**Figure 4:**
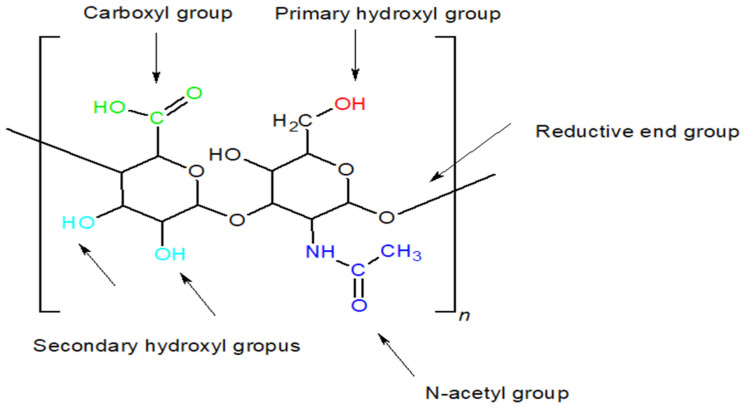
Reactive functional groups of HA (illustration based on [107]).

HA can undergo chemical modifications to enhance its physicochemical properties and generate derivatives that enable its application as a drug carrier while preserving the natural attributes of the compound, such as biocompatibility and biodegradability [105,106]. These modifications are achieved through methods including deacetylation, esterification, and amidation of the acid’s functional groups Reactive functional groups og HA are presented on Figure 4.

#### 5.1.1. Modifications of the –OH Group

**Figure 5:**
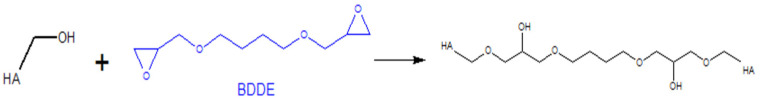
The reaction of HA with BDDE under alkaline conditions.

**Figure 6:**
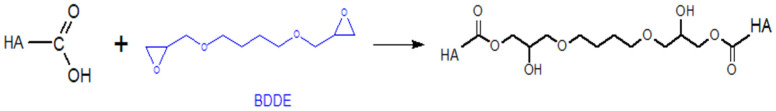
The reaction of HA with BDDE under acidic conditions.

Each disaccharide unit contains four hydroxyl groups: one primary hydroxyl group at the C6 carbon of the *N*-acetylglucosamine residue and three secondary hydroxyl groups. These functional groups constitute the principal reactive sites in esterification and etherification processes [105,108]. Hydroxyl groups can react with epoxides, leading to the formation of cross-linked HA. A popular compound used in this reaction is 1,4-butanediol diglycidyl ether (BDDE) [105,108,109]. The reaction proceeds under alkaline conditions, where most –OH groups are deprotonated and thus become more nucleophilic. The opening of the epoxide ring results in the formation of ether linkages with the hydroxyl groups. If the reaction is carried out in an acidic environment, with a pH lower than the pKa of the –OH group, bond formation with the carboxyl group will occur [105,108]. The reaction pathways under alkaline and acidic conditions are presented in Figure 5 and Figure 6, respectively.

**Figure 7:**
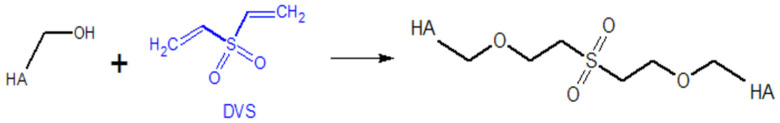
The cross-linking reaction of HA using DVS.

The cross-linking reaction of HA using divinyl sulfone (DVS) (Figure 7) proceeds at pH > 13 and room temperature [105,108]. Conducting the reaction at ambient temperature limits acid depolymerisation compared to methods employing significantly higher temperatures [108]. This reaction leads to bis(ethylsulfonyl) ether linkages between the hydroxyl groups of HA.

**Figure 8:**
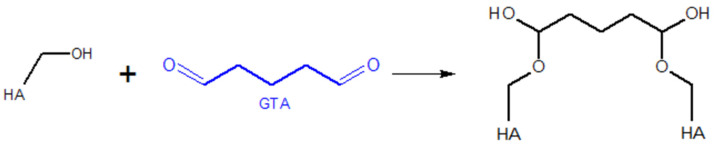
The reaction of HA with GTA.

The reaction of HA with glutaraldehyde (GTA) leads to the formation of hemiacetal bonds. This reaction is conducted in an acetone–water environment at pH = 2. The acidic environment favours aldehyde activation, initiating the reaction [105,108]. The course of this reaction is presented in Figure 8.

**Figure 9:**
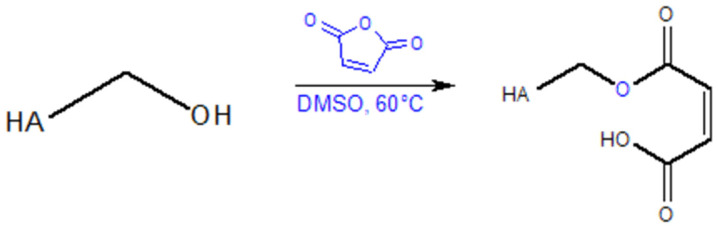
The reaction of hydroxyl groups with maleic anhydride.

Vasi et al. [109] describe the synthesis of a hyaluronic acid (HA) derivative through the reaction of hydroxyl groups with maleic anhydride (MA). MA, being a highly reactive anhydride, facilitates the formation of non-toxic compounds via a ring-opening mechanism in an organic solution (HA dissolved in DMSO at 60 °C). The reaction of hydroxyl groups with maleic anhydride is shown on Figure 9.

This chemical modification enabled the creation of a novel derivative, which was subsequently employed in formulating hydrogels with a defined pore structure. The hydrogel was fabricated through the copolymerisation of acrylic acid in the presence of a redox initiation system. The resulting gels were evaluated for their drug release profiles and cytotoxicity. The findings indicate their potential for applications involving contact with biological environments [109].

#### 5.1.2. Modifications of the –COOH Group

Molecular modelling demonstrates that hyaluronic acid (HA) carboxyl groups are well-established recognition sites for both cell surface receptors CD44 and the enzymes Hyal-1 and Hyal-2. Consequently, chemical modification of the –COOH groups is anticipated to significantly impact its biological activity within the organism [108,109,110]. A single carboxyl group is present within each disaccharide unit, located on the D-glucuronic acid residue, which is susceptible to modification via esterification or amidation.

**Figure 10:**
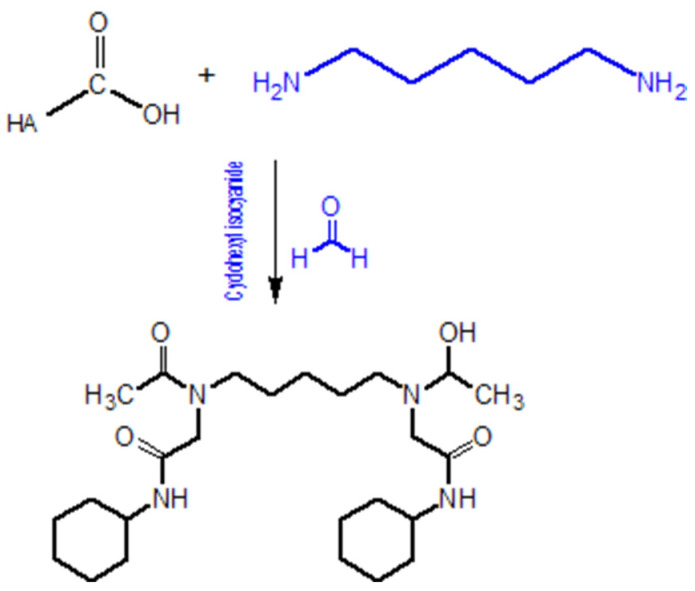
Ugi condensation reaction.

The Ugi condensation reaction (Figure 10), employing diamines as the amine component, can cross-link hyaluronic acid (HA) chains by forming amide bridges. This approach enables the synthesis of hyaluronic acid hydrogels with controlled mechanical and degradation properties. The reaction utilises formaldehyde, cyclohexyl isocyanide, and a diamine. The process is conducted in an acidic aqueous environment. Formaldehyde reacts with the diamine to form a protonated imine, which subsequently reacts with cyclohexyl isocyanide. As a result of the reaction, the carboxylic acid group of HA forms an amide bond with the protonated diamine [111].

**Figure 11:**
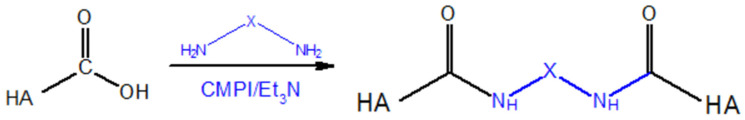
Reaction of amidation.

The amidation reaction (Figure 11) employing 2-chloro-1-methylpyridinium iodide (CMPI) in dimethylformamide (DMF) results in the activation of carboxyl groups, rendering them susceptible to nucleophilic attack. This reaction can proceed effectively if the carboxylic acid (HA) is initially converted to its tetrabutylammonium (TBA) salt to facilitate dissolution in the organic solvent. Literature precedent [108] indicates that CMPI reacts with the –COOH group, forming a pyridinium intermediate. Subsequently, the amide bond is formed with the activated carboxyl group.

**Figure 12:**
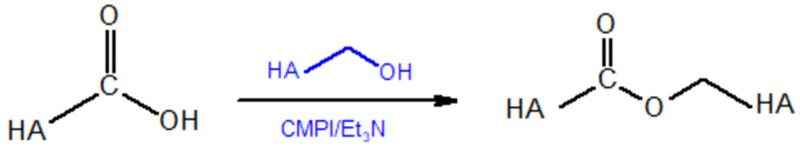
Reaction of ester formation.

In a basic environment, the activated carboxyl groups are susceptible to nucleophilic attack by –OH groups present in other segments of the HA chain. This phenomenon leads to self-crosslinking through the formation of intermolecular ester bonds [105,108]. Due to the nature of the organic reagents utilised, purification of the resulting compound is a necessary step. Reaction of HA ester formation is presented on Figure 12

**Figure 13:**
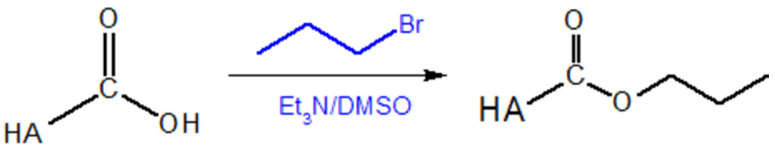
Reaction of HA with an alkyl halide.

The application of alkyl halides leads to the formation of esters through the –COOH group, reducing the negative charge on the HA molecule. The introduction of alkyl chains also influences the modified acid’s hydrophobicity. In solution, self-assembly and the formation of micelles or nanoparticles may occur. Organic solvents are frequently employed, necessitating the conversion of HA to TBA-HA to enhance solubility. The cours e of reaction of HA with an alkyl halide is presented on Figure 13.

**Figure 14:**
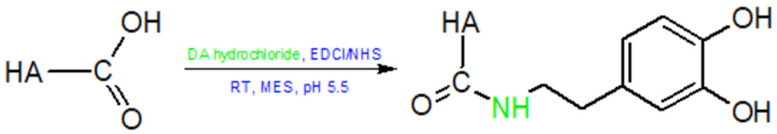
Reaction of HA with DA.

As a result of the chemical modification of HA, specifically the amidation of the carboxylic acid group with a primary amine derived from a dopamine (DA) molecule, a novel material was obtained [112]. The pathway of this reaction is presented on Figure 14.

The resulting HA-DA hydrogel exhibits the capacity for self-crosslinking through dopamine autoxidation. Due to enhanced modifications, the HA-DA hydrogel can undergo in situ gelation and firmly adhere to the intestinal surface due to enhanced bioadhesion. Upon hydrogel formation within the intestine, a robust physical barrier is created, which aims to prevent the invasion of pathogenic bacteria into the intestinal mucosa. Furthermore, HA-DA promoted the proliferation and migration of intestinal epithelial cells, indicating its potential to accelerate wound healing and epithelial regeneration. This gel holds promise for treating inflammatory bowel diseases (IBD) [112].

#### 5.1.3. Modifications of the NHCOCH3 Group

Modification of the *N*-acetyl group necessitates prior deacetylation. The resulting amino group can subsequently undergo reactions to form amide bonds. *N*-deacetylation can be performed using enzymatic or chemical methods [113,114,115].

**Figure 15:**
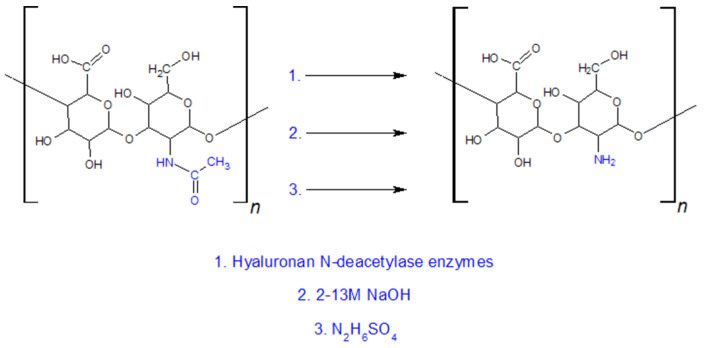
Ways of HA N-deacetylation.

Chemical N-deacetylation typically employs strong bases [114] or acids [115] and elevated temperatures (55–105 °C). Under such conditions, in addition to *N*-deacetylation, hydrolytic cleavage of glycosidic bonds also occurs, leading to extensive depolymerisation of hyaluronic acid (HA) [113,114,115]. Sodium hydroxide has begun to be replaced by hydrazine sulphate. The carboxyl groups present in the HA structure are partially converted to hydrazide groups under the influence of hydrazine, which can then be converted back to carboxyl groups [113]. Enzymatic deacetylation proceeds under mild conditions and without depolymerisation of the HA chain. The enzymes responsible for these reactions are hyaluronan N-deacetylases [113]. Ways of HA N-deacetylation are presented on Figure 15.

### 5.2. Physical and Chemical Hydrogels Creation

#### 5.2.1. Physical Crosslinking

Physical crosslinking involves forming a three-dimensional network by associating HA chains via physical interactions, such as hydrogen bonds, ionic, Van der Waals, hydrophobic, or electrostatic interactions. This approach allows for creating structures with low toxicity and straightforward implementation. At the same time, we obtain gels with low mechanical strength and structural stability [116,117]. Examples of physical crosslinking are described below.

#### 5.2.2. Coordination Crosslinking

The hydrogel structure can be formed through the coordination crosslinking of metals with negatively charged, ionisable functional groups of HA. Importantly, these interactions enable the formation of hydrogels without the necessity for chemical modification of the polymer [118]. The study authors (G. Prakash et al.) utilised divalent metal ions such as Mn(II), Fe(II), Co(II), Ni(II), Cu(II), Zn(II), Pd(II), and Mg(II). As a result of alkalising the environment with sodium hydroxide, HA undergoes deprotonation, leading to an increase in negative charges on the chains, favouring interaction with positively charged metal cations and promoting hydrogel formation. The main factors that allow for the modification of the properties of the produced gels, particularly the rheological and viscoelastic properties, are the molecular weight of HA, the concentration of metal ions, the concentration of HA, the concentration of sodium hydroxide, and the ionic radius of the divalent cation. It was observed that smaller ions more frequently crosslink HA, which manifests as an increase in the storage modulus. Gels crosslinked with Mg(II) exhibited high stability; self-healing potential; responsiveness to stimuli (specifically, salt-responsivity); and unique physicochemical properties, including conductivity and antibacterial properties [118].

#### 5.2.3. Chemical Crosslinking

Chemical crosslinking involves the formation of covalent bonds between HA chains. This reaction results in stable, three-dimensional networks. Chemical hydrogels are formed through polymer crosslinking via irradiation, chemical crosslinkers, or polyfunctional compounds. Consequently, these systems exhibit enhanced chemical, mechanical, and thermal stability compared to physical hydrogels [108,116]. Examples of chemical crosslinking reactions are described below.

##### Carbodiimide Crosslinking

**Figure 16:**
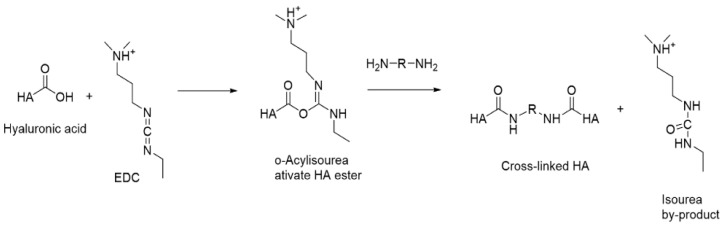
Carbodiimide crosslinking reaction [117].

**Figure 17:**
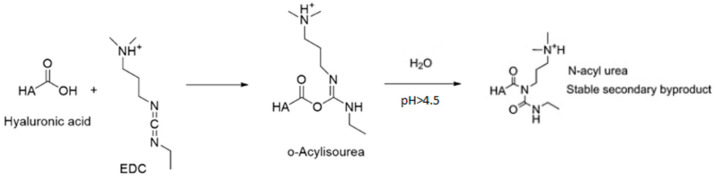
Formation of an amide product (based on [108,117]).

Ethyl-3-(3-dimethylaminopropyl) carbodiimide (EDC) is frequently employed to facilitate HA crosslinking (Figure 16). EDC acts as a coupling agent, promoting the formation of covalent bonds through the activation of carboxyl groups; however, the compound itself is not incorporated into the structure of the crosslinked hydrogel. The reaction proceeds between a carboxyl group and primary amines, which function as nucleophiles, in a slightly acidic environment at room temperature [105,108]. Initially, an intermediate compound, O-acylisourea, is formed. The resulting reaction products are contingent upon the pH of the solution. Under acidic conditions (pH 3.5–4.5), the activation of the carboxylic acid by EDC occurs. Conversely, in an environment with a higher pH, the amine undergoes deprotonation, leading to the formation of an amide. Formation of an amide product pathway is presented on Figure 17.

The highly reactive *O*-acylisourea readily reacts with water and rapidly converts into a stable byproduct, *N*-acylurea [108].

##### Diisocyanate Crosslinking

The advantage of employing isocyanates in crosslinking reactions lies in their reactivity. The reaction results in the formation of urethane linkages between the hydroxyl groups present in the HA structure and the isocyanate [105,119]. F. Zamboni et al. [120] developed bis(β-isocyanatoethyl) disulfide (BIED), the structure of which is based on alkyl groups rather than the aryl groups found in conventional diisocyanates. The resulting gels exhibited greater stability and underwent slower hydrolytic degradation. An important characteristic of the crosslinking agent used is that alkyl isocyanates are not cytotoxic, unlike aryl isocyanates. The obtained gels retained biocompatibility [119].

##### Michael Addition

This process employs crosslinking agents containing thiol groups, acting as Michael donors. Thiol groups are strong nucleophiles and react with Michael acceptors, which are acrylate groups in the modified HA molecule. Methacrylate-modified HA (MeHA) is obtained through the esterification of the hydroxyl group of the *N*-acetyl-d-glucosamine unit with methacrylic anhydride. The reaction proceeds in an environment with a pH of 8–9 [121].

##### Diels–Alder Reaction

The Diels–Alder reaction is classified as a “click” reaction. It proceeds under mild conditions, generates easily removable byproducts, and is additionally fast and selective. This cycloaddition reaction must be preceded by the modification of the HA molecule to introduce appropriate functional groups. For this purpose, the carboxyl group of HA undergoes an amidation reaction. As a result of the introduced changes in the molecule’s structure, the reaction occurs between furan, acting as a diene, and maleimide, acting as a dienophile [116,117,122].

##### Host–Guest Complexes

Host–guest complexes are formed through a process where one molecule (the guest) is trapped within the crystal lattice of a second, larger molecule (the host) [120]. The interactions maintaining the complex are weaker than covalent bonds. The interactions present within the molecular structure include Van der Waals forces, hydrogen bonds, or hydrophobic interactions. The most popular host molecule is cyclodextrin.

##### Gelling Agents

Pluronic-based gels can be utilised as injectable gels; however, they exhibit limited stability and undergo rapid dissolution under physiological conditions [120]. The physical blending of high molecular weight HA with Pluronic F-127 in an aqueous solution enabled the development of a novel thermosensitive HP hydrogel. Within the HP structure, HA molecules function as intermolecular crosslinkers for the micelles, resulting in the formation of a remarkably dense micellar structure at temperatures below the critical gelation temperature (CGT). The achievement of such a compact gel structure holds potential for application as a drug carrier for sustained release. Furthermore, the incorporation of HA influenced a reduction in the CGT compared to native Pluronic F-127. This lower sol-to-gel transition temperature favourably impacts the in vivo application of HP, which represents a crucial characteristic for injectable gels [123].

##### Functionalisation of HA with Hydrophobic Molecules

Hydrophobically modified HA (HPHA) is a derivative of HA in which hydrophobic moieties are covalently attached to the hydrophilic polysaccharide backbone. These hydrophobic groups encompass fatty acid residues, long alkyl chains, cholesterol, hydrophobic drugs, or synthetic polymer blocks. The introduction of hydrophobic groups to the highly hydrophilic HA backbone results in the formation of amphiphilic macromolecules [124]. In aqueous environments, HPHA self-assembles into micelles, nanoparticles, and physical hydrogels driven by the aggregation of hydrophobic domains. HPHA can be synthesised via esterification, where hydroxyl groups are esterified with acid anhydrides, acid chlorides, and activated carboxylic acids, while carboxyl groups undergo reactions with hydrophobic alcohols or epoxides. Furthermore, hydrophobic amines can be conjugated to carboxyl groups through amidation [107]. An alternative strategy for creating amphiphilic HA derivatives involves the attachment of pre-formed hydrophobic polymer blocks to the HA backbone. Polymers such as PLA, PCL, or PHA can be covalently linked to the HA scaffold. Grafting pre-formed polymer blocks allows for more precise control over the length of the hydrophobic segment.

##### Crosslinking via Condensation Reactions

A condensation reaction involves the formation of a covalent bond with the simultaneous elimination of a small molecule, most commonly water. The most prevalent method for HA crosslinking is the previously described condensation utilising carbodiimides. We can highlight three types of condensation reactions: aldol condensation, Schiff base formation, and andoxime formation.

Aldol condensation for HA crosslinking leads to the formation of stable C-C bonds. Hydrogels synthesised through this method exhibit excellent hydrolytic stability and favourable mechanical properties. The aldehyde groups present in the aldol product impart adhesive properties to the hydrogels towards tissues [125].

Schiff bases are the products of the condensation of primary amines and carbonyl compounds (aldehydes or ketones). The reaction results in the formation of imines and water as a byproduct. Native HA lacks carbonyl groups, necessitating chemical modification. The most common method involves oxidation using NaIO_4_, which cleaves C-C bonds in the glucuronic acid units, generating reactive aldehyde groups. The resulting oxi-HA can then be crosslinked with polymers containing amine groups, such as chitosan [126].

An oxime linkage is formed through the condensation of a carbonyl group with a hydroxylamine group. This is a “click”-type reaction that is bioorthogonal, as the two reactants react efficiently and specifically with each other in the presence of other functional groups. The oxime bond formation reaction is acid-catalysed. Lower pH values accelerate hydrogel formation. HA requires modification to introduce aldehyde groups. Crosslinking occurs via reactions with aminooxy-polyethylene glycol (AO-PEG) containing hydroxylamine groups [127].

##### Radical Polymerisation

**Figure 18:**
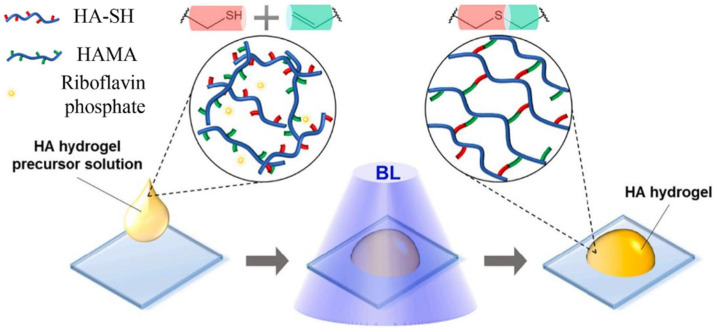
A diagram of photoinitiated polymerisation, using polymerisation of HA-SH and HAMA as an example [122].

Radical polymerisation is caused by free radicals that get released due to decomposition of an initiator compound when it is affected by heat, light, or oxidative reactions. The free radicals initiate polymerisation of monomers which contain specific functional groups. This approach for creating hydrogels has many advantages, including high efficiency and being able to tune the mechanical properties of the product. Polymerisation initiated with the use of light is a great method for manufacturing of HA-based hydrogels. The main problem in this method is that some photoinitiators can be toxic [122]. Schematic representation of photoinitiated polymerization, using the polymerization of HA-SH and HAMA is shown on Figure 18.

##### Enzymatic Crosslinking

**Figure 19:**
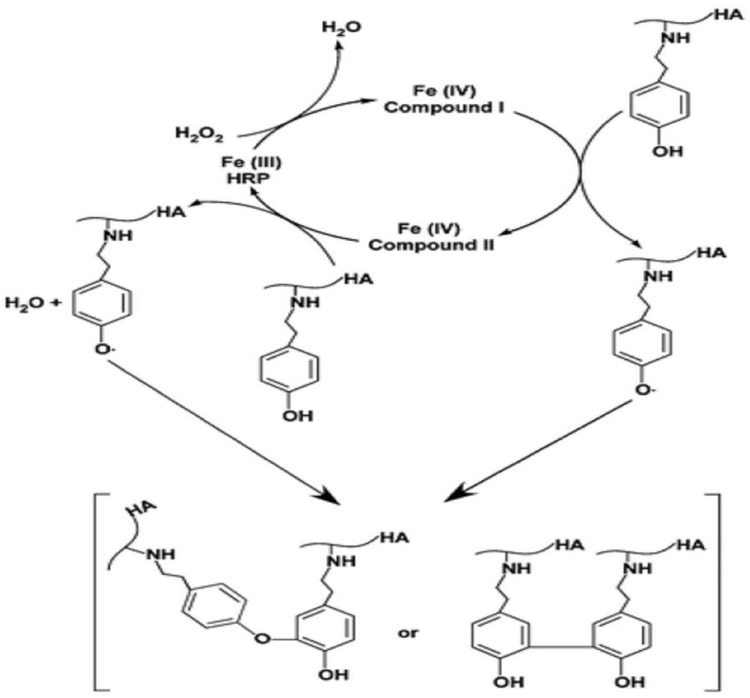
A diagram of HA-Tyr crosslinking reaction catalysed by HRP [122].

Enzymatic catalysis can activate specific groups in HA, which causes chemical crosslinking. This method of preparing HA hydrogels is useful for creating injectable hydrogel material, thanks to causing rapid gelation under mild conditions. An example of enzyme used in hydrogel preparation would be horseradish peroxidase (HRP). It is a single chain protein that, in the presence of H_2_O_2_, catalyses coupling of aniline and phenol derivatives. HRP-catalysed reaction can be used in crosslinking of HA functionalised with Tyr (Figure 19). In this system, the mechanical strength of the gel and the rate of gelation can be controlled by adjusting the concentration of H_2_O_2_ and HRP [122,128].

##### Thiol-Ene Photocoupling

**Figure 20:**
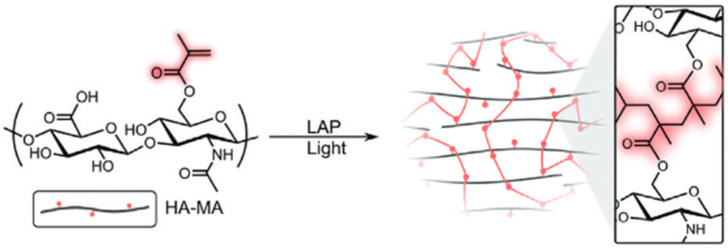
Schematic of formation of a thiol-ene hydrogel [129].

The thiol-ene reaction results in formation of a thioether, as a result of addition of a thiyl radical to a vinyl group. The resulting compound is a photocrosslinkable and can be used in hydrogel manufacturing. This methods advantage over radical polymerisation is that it is not sensitive to the presence of oxygen [129]. Figure 20 illustrates the formation process of a thiol-ene hydrogel.

##### Disulfide Crosslinking

**Figure 21:**
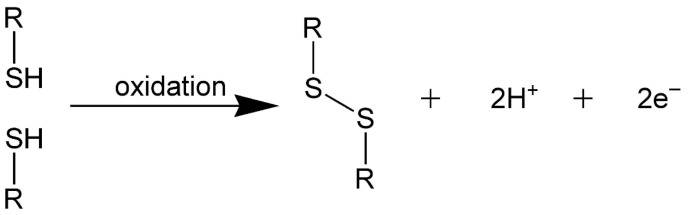
A schematic of a disulfide bond creation [132].

A disulfide bond is a covalent bond, usually created between two coupled thiol groups, which undergo an oxidation reaction (Figure 21). In HA-based hydrogels, HA needs to be sulfurised for crosslinking with other compounds containing thiol groups [130,131].

Table 4 summarizes the properties of hydrogels formed using various crosslinking techniques.

### 5.3. HA-Based Hydrogels

#### 5.3.1. HA Hydrogels

HA hydrogels have many applications in the biomedical field thanks to their excellent biocompatibility, including uses in tissue engineering, drug delivery, cancer therapy, and wound healing. The properties of HA hydrogels, like their molecular weight and modification of functional groups, impact their function and use. Sulfonation is an essential method of HA modification, as it can improve its biological functions. HA can also be coupled with other substances, like chitosan, gelatin, and alginate [81,133]. Hydrogels composed solely of HA have not been extensively studied, primarily due to the high cost of HA and the complexity of its crosslinking. V. Dulong and colleagues proposed a hydrogel based on HA crosslinked with trisodium trimetaphosphate (STMP). A key feature of STMP is its low toxicity and the absence of adverse effects on the human body. The crosslinking reaction occurs through the polysaccharide’s hydroxyl (–OH) groups, leading to the formation of ester bonds. Additionally, the response with STMP introduces anionic charges via phosphate groups. The particle size was generally highly heterogeneous, ranging from 5 to 200 µm. This variability can be attributed to the synthesis method, particularly to the irregular introduction of the viscous HA solution into the reactor, which was hindered by the high viscosity of the solution. Additional factors contributing to this heterogeneity may include the stirring speed, the viscosity of the organic phase, and the choice of mixing geometry [134].

#### 5.3.2. Ha-Col Hydrogels

Collagen (Col) is a protein found in bone tissue. The combination of Col with HA hydrogels has been a research subject for use in bone tissue engineering. Col addition can improve HA hydrogels’ mechanical properties while promoting cell adhesion and proliferation. HA-Col hydrogels have been proven to enhance bone regeneration [135]. In 2019, Huiyan et al. developed a COL-HA hydrogel via in situ coupling of phenolic groups from collagen I–hydroxybenzoic acid conjugate (COL-P) and hyaluronic acid–tyramine (HA-Tyr), catalysed by horseradish peroxidase (HRP). The resulting COL-HA hydrogel exhibited a porous structure, facilitating gas exchange and the diffusion of nutrients and culture medium. This material was designed for application as a wound dressing hydrogel [136].

#### 5.3.3. HA-Alg Hydrogels

Alginates (Alg) are linear polysaccharides. HA-Alg hydrogels have been found to be useful in regenerative medicine as a material for 3D bioprinting. Adding HA to the Alg hydrogel promotes cell migration and wound healing, while Alg provides structural stability. This hydrogel formulation can also be a potential platform for other substances [135]. HA-Alg hydrogels are also promising drug carriers for antibiotic delivery. In 2023, Trusek et al. developed a novel biphasic HA/Alg drug carrier coated with a polylactide layer. Coating the carriers with polylactide increased the release time by around forty times. As the carriers were designed to reduce local bacterial infections, among others in dentistry, the released antibiotics were amoxicillin, metronidazole, and doxycycline [137]. In 2013, Min-Dan Wang and colleagues developed a hydrogel based on hyaluronic acid (HA) combined with alginate. HA modified with adipic acid dihydrazide (ADH) was reacted with sodium alginate. Calcium chloride was used as the crosslinking agent for alginate, while 1-ethyl-3-(3-dimethylaminopropyl)carbodiimide (EDC) was employed to crosslink the HA. Changes in absorbance following the addition of ADH indicated the formation of an increased number of amide bonds, consistent with the mechanism of carboxyl–amine coupling facilitated by EDC. Morphologically, the crosslinking of HA with ADH in EDC resulted in hydrogels with improved mechanical strength compared to those without ADH. The hydrogel was used to fabricate scaffolds via 3D printing. These scaffolds exhibited biocompatibility with Schwann cells and supported their survival and growth [138].

#### 5.3.4. HA-CS Hydrogels

Chitosan (CS) is a chitin-based bioactive polymer. In hydrogels, CS acts as a structural component, improving mechanical properties and promoting cell proliferation. HA-CS hydrogels have been shown to impact bone regeneration positively [139]. In 2017, a research team from Nanjing University led by Youming Deng developed an HA-CTS hydrogel to support abdominal tissue regeneration. For this purpose, they employed hyaluronic acid aldehyde (AHA) and N, O-carboxymethyl chitosan (NOCC). AHA was synthesised by oxidising HA using sodium periodate (NaIO_4_), which converted adjacent hydroxyl groups into dialdehydes, opening the sugar ring and generating dialdehyde derivatives. The CTS-HA hydrogel was formed by crosslinking NOCC with AHA. The gelation time ranged from 70 to 2400 s and was significantly shortened with increasing AHA concentration. At a molar ratio of 1:2 (NOCC: AHA), gelation occurred in under two minutes. The CTS-HA hydrogels were formed in situ via a Schiff base reaction. In vivo, the hydrogel promoted abdominal tissue formation, and cytokines involved in angiogenesis and the corresponding cellular responses were also positively modulated [140].

#### 5.3.5. HA-Gel Hydrogels

Gelatin (Gel) is a result of denaturing collagen. Similarly to Col, it can be used as a structural component, improving physiochemical attributes of hydrogels and promoting cell proliferation and growth. The properties of HA-Gel hydrogels allowed them to be used in tissue engineering [139]. Poveda-Reyes et al. designed an HA/Gel hydrogel system composed of gelatin and hyaluronic acid conjugated with tyramine, using horseradish peroxidase as a crosslinking agent. The Gel and HA chains were modified by coupling their carboxyl groups with tyramine moieties, employing EDC and NHS as coupling agents. The crosslinked Gel/HA hydrogels were then obtained through covalent bonding of the tyramine groups via horseradish peroxidase and hydrogen peroxide (H_2_O_2_). For all Gel/HA combinations containing both components, successful myotube formation was observed throughout the hydrogel without contraction, indicating the potential of these systems as promising candidates for skeletal muscle or soft tissue engineering. It was also found that increasing the HA content within the hydrogel formulation led to higher Young’s moduli [141].

#### 5.3.6. HA-PCL Combinations

Polycaprolactone (PCL) is a biocompatible polyester with broad application in the biomedical field. Combining PCL scaffolds with HA hydrogels is a promising strategy for developing scaffolds that can provide an appropriate environment for tissue regeneration and act as carriers for bioactive agents [139]. HA-PCL copolymers have also been researched as carriers for a targeted drug delivery system for delivering drugs to the brain [142].

## 6. Conclusions

Hyaluronic acid stands at the forefront of biomaterial innovation, offering a unique combination of biological relevance and structural versatility. Advances in its production—particularly through microbial fermentation and chemical modification—have opened new avenues for customising HA’s physicochemical properties to suit diverse biomedical applications. Numerous studies have shown that the molecular weight of hyaluronic acid (HA) can be effectively modulated by selecting specific culture media and microbial strains. This controllability allows HA to be tailored for particular applications, further enhancing its versatility and desirability as a biomaterial. In parallel, progress in crosslinking strategies has enabled the development of HA-based hydrogels with finely tuned mechanical strength, degradation kinetics, and bioactivity. These hydrogels are highly effective platforms for localised and sustained drug delivery, demonstrating potential in oncology, regenerative medicine, ophthalmology, and wound healing. Despite these promising developments, several challenges remain, not only during the production stage (an increase in viscosity, mixing and aeration problems) but also in achieving cost-effective large-scale production, enhancing batch-to-batch consistency, and meeting regulatory standards for clinical application. Ongoing interdisciplinary research and collaboration between materials scientists, biologists, and clinicians will be essential to harness HA’s capabilities fully.

## Figures and Tables

**Table 1:** Network formation and viscosity behaviour by HA molecular weight [10].

MW Range	Network Formation	Viscosity Behavior	Stability
60–90 kDa	None (rigid rods)	Newtonian (no shear-thinning)	High thermal and enzymatic stability
~200 kDa	Intermediate	Shear-thinning at high concentration	Moderate stability
400–2500 kDa	Strong Entanglement	Pseudoplastic (shear-thinning)	Lower stability, depolymerisation more likely

**Table 2:** Main differences in HA production methods [37].

Production Method	Key Characteristics	Advantages	Limitations
Animal tissue Extraction	Extraction via chemical and enzymatic digestion (e.g., papain, trypsin)	High-molecular-weight HA; widely available tissues	Risk of pathogens; batch variability; ethical concerns
Bacterial Fermentation	Fermentation in bioreactors using glucose or other carbon sources; produces capsular HA	Scalable; “vegan” HA; cost-effective	May contain endotoxins; requires downstream purification
Cell-free In vitro systems	Uses isolated HAS enzymes in a cell-free system to synthesise HA	High specificity; potential for defined Mw products	Currently limited to research scale; complex enzyme stabilisation; low yield

**Table 3:** Comparison of HA from different sources.

Production Method	Source	Molecular Weight [Da]	Yield	Advantages	Disadvantages	References
From animal tissues	Rooster combs	1.2 × 10^6^	6.00%	Well-established technology	Product quality variability	[75]
	Pig umbilical cord	3.4 × 10^6^	4.40%	Low-cost and accessible raw material	Risk of polymer degradation	[76]
	Bovine vitreous body	7.7 × 10^4^–1.7 × 10^6^	0.12%	High molecular weight product	Risk of contamination (e.g., proteins)	[77]
	Bovine synovial fluid	1.4 × 10^6^	0.0037%	Natural product	Requires extensive purification, very low yield	[78]
Fermentation	*Streptococci*	2.1 × 10^6^–7.4 × 10^7^	0.44–6.94 g/L	High yield	Use of pathogens and GMOs	[79]
	*Streptococcus thermophilus*	2 × 10^6^	1.2 g/L	Optimised technology	Risk of endotoxin contamination	[79]
	*Bacillus subtilis*	1–1.2 × 10^6^	5 g/L	High molecular weight product	Contamination risk (proteins, heavy metals)	[80]
	*Lactococcus lactis*	0.4–4.1 × 10^6^	0.65 g/L	Food-grade organism; potential for genetic engineering	Lower yield; requires metabolic pathway optimisation	[9,66]
	*Escherichia coli*	1.5 × 10^6^	3.8 g/L	Well-characterised genetics; rapid growth; scalable fermentation	Endotoxin contamination risk; requires extensive purification	[43]
	*Pichia pastoris*	8 × 10^2^–2.5 × 10^6^	20–90%	High yield; scalable; suitable for pharmaceutical applications	Produces low molecular weight HA; emerging technology	[37]
In vitro synthesis	—	8 × 10^2^–2.5 × 10^6^	20–90%	Versatile technology; free from contamination; stable product quality;	Emerging and still developing technology; not economically viable	[81]

**Table 4:** Comparison of hydrogel characteristics obtained through different crosslinking methods.

	Physical Crosslinking	Chemical Croslinking	References
Mechanism	Non-covalent interactions: hydrogen bonds, electrostatic interactions, metal coordination	Covalent bonds: carbodiimide crosslinking, diisocyanate crosslinking, Schiff base crosslinking	[107,119,121,122,129,130,131]
Crosslinking agents	Does not require the use of chemical crosslinking agents	Requires the use of chemical compounds that react with functional groups (e.g., EDC)	[119,121,122,124,129,130,131]
Method difficulty	Simple method, does not require complex chemical reactions; does not require the removal of byproducts	Requires precise selection and concentration of the crosslinking agent, control of reaction conditions; requires the removal of unbound crosslinking agent residues and potential byproducts	[107,119,121,129,130,131]
Biocompatibility	Preservation of natural biocompatibility	Biocompatibility depends on the type and amount of crosslinking agent used and the effectiveness of its removal after the crosslinking process	[107,119,121,129,130,131]
Durability	Gels are less durable, susceptible to environmental changes	Gels are more stable anddurable, resistant to rapid enzymatic degradation	[107,119,121,129,130,131]
Mechanical properties	May have lower mechanical strength and structural stability	Allows for obtaining structures with a wide range of mechanical properties, i.e., high viscosity, elasticity, ability to support tissues	[107,119,121,129,130,131]
Porosity	Difficult to control; structures with larger pores	Enables control; allows for the design of structures with specific properties	[107,119,121,129,130,131]
Rheological properties	Exhibit shear-thinning behavior; recovering viscosity after shear removal	It is possible to obtain gels with the desired viscosity and storage modulus	[107,119,121,129,130,131]

## Data Availability

No new data were generated in this review article. All studies and data reported are available publicly from the references cited.
